# Exploring medical first responders’ perceptions of mass casualty incident scenario training: a qualitative study on learning conditions and recommendations for improvement

**DOI:** 10.1136/bmjopen-2024-084925

**Published:** 2024-07-11

**Authors:** Fredrik Schulz, Quynh Nguyen, Anke Baetzner, David Sjöberg, Lina Gyllencreutz

**Affiliations:** 1 Department of Nursing, Umeå University, Umea, Sweden; 2 Department of Diagnostics and Intervention, Umeå University, Umea, Sweden; 3 Center for Technology Experience, Austrian Institute of Technology GmbH, Wien, Austria; 4 Department for Artificial Intelligence and Human Interfaces, Paris Lodron University of Salzburg, Salzburg, Austria; 5 Institute of Sports and Sports Sciences, Heidelberg University, Heidelberg, Germany; 6 Unit of Police Work, Umeå University, Umea, Sweden

**Keywords:** qualitative research, nursing care, virtual reality, trauma nursing, medical education & training

## Abstract

**Abstract:**

**Objective:**

Despite participating in scenario training, many medical first responders (MFRs) perceive themselves as inadequately prepared to respond to mass casualty incidents (MCIs). The objective of this study was to conduct a comprehensive examination of traditional MCI scenario training methods, focusing on their inherent strengths and limitations. An investigation into the perceptions of MFRs who had participated in MCI scenario training was carried out to identify potential areas for improvement and provide recommendations for refining MCI training protocols.

**Design:**

Qualitative inductive approach using semistructured interviews that took place between October 2021 and February 2022. Data were analysed with qualitative content analysis.

**Setting:**

MCI scenario training involving four organisations (three emergency medical services and one search-and-rescue organisation) tasked with responding to MCIs, collectively representing four European Union countries.

**Participants:**

27 MFRs (17 emergency medical services personnel and 10 search-and-rescue volunteers) were recruited to participate in the study.

**Results:**

Two categories and seven associated subcategories (shown in parentheses) were identified as influencing the learning outcomes for MFRs: Training in a context mirroring real-world incidents (conducting incident scene risk assessment, realistic representation in casualties, incorporating scenario variety into the curriculum, interagency collaboration, role alignment when training incident site management) and use of a pedagogical framework (allowing for mistakes, the importance of post-training evaluation).

**Conclusions:**

This study reaffirms the value of traditional MCI scenario training and identifies areas for enhancement, advocating for realistic scenarios, interagency collaboration, improved incident site management skills and thorough post-training evaluation. It suggests a shift in MCI training conceptualisation and delivery. The potential of virtual reality technologies as a valuable addition to training methods is explored, with a note on the need for further research to ascertain the long-term effectiveness of these technologies. However, the selection of a training method should consider programme goals, target population and resources.

**STRENGTHS AND LIMITATIONS OF THIS STUDY**

- Conducting semistructured interviews immediately following the mass casualty incident (MCI) scenario training enabled the capture of the medical first responders’ most recent experiences with traditional MCI scenario training.
- Incorporating both emergency medical service personnel and search-and-rescue volunteers into the study allowed for a more comprehensive understanding of the perspectives of medical first responders, enriching the insights into traditional MCI scenario training.
- Employing interpreters for some interviews may have introduced inconsistencies in question phrasing and comprehension, potentially influencing the responses and thereby affecting the data collected.
- The heterogeneity of the sample, characterised by differences in education, training and previous experience among the participants, introduces a degree of variability in the data that presents a limitation in interpreting the findings due to the diverse backgrounds of the participants.

## Introduction

 In the domain of emergency response, the occurrence of mass casualty incidents (MCIs) presents significant challenges for medical first responders (MFRs).^1 2^ MFRs are defined as personnel who have received specialised training to respond to MCIs and are competent in providing immediate medical intervention and assistance in the affected area. The role encompasses both emergency medical service personnel (EMS) and trained volunteers.^3^ Characterised by an overwhelming number of casualties that surpass the immediate resources available for their care, MCIs demand a level of preparedness and proficiency that extends beyond routine medical practice.^4^ The unique complexities associated with these situations, including the sudden surge in patient volume and the inherent unpredictability of injuries and resource allocation, underscore the need for a specialised skill set and strategic approach.^5^

The multifaceted demands of disaster response require a skill set extending beyond routine medical practice, illuminating the intricate and complex nature of the MFR role in managing the challenges posed by MCIs.^4 6^ Responders must address a diverse range of injuries, from minor to life-threatening, demanding swift decision-making in resource allocation and appropriate care. This underscores the imperative for adaptability and highlights the essential need for MFRs to demonstrate proficiency in strategic coordination, rapid decision-making, effective communication and resource allocation.^7^

Beyond the foundational qualifications necessary for their roles, additional training is a pivotal in preparing MFRs for disaster response. Within the European Union (EU), each member state is individually responsible for organising disaster response and implementing disaster response training programmes.^8 9^ As MCIs are predominantly infrequent events, many MFRs lack opportunities to learn from experiencing real-word catastrophic events firsthand.^10^ To emulate real-world MCI conditions, live full-scale scenario training is widely recognised as the benchmark for disaster preparedness. This approach aims to afford MFRs the opportunity to acquire and hone core competencies crucial for managing large-scale incident scenes.^1 11 12^

Despite its status as a gold standard, research indicates notable challenges associated with full-scale MCI scenario training, impinging on its efficacy.^13^ Organisational constraints, encompassing budgetary limitations, resource allocation hurdles and the demanding healthcare sector workload, pose substantial barriers to executing comprehensive training. These challenges restrict the active participation of MFRs and limit the focus on thorough post-training evaluations essential for assessing training effectiveness.^13^ Furthermore, prior studies have underscored that the organisational structure of full-scale MCI scenario training often falls short in providing participants adequate time for analysis and discussion, hindering their ability to identify weaknesses or explore alternative solutions.^10 14 15^

Consequently, these challenges culminate in a training experience that may fall short of the desired authenticity, leaving MFRs without the practical exposure and confidence crucial for managing unforeseen catastrophic events. The resultant sense of unpreparedness reported among healthcare professionals^216^,^20^ underscores the pressing need for a rigorous examination of current MCI scenario training methods. Through a detailed examination of factors that promote learning and the inherent limitations in current training strategies, the intent is to uncover potential areas for improvement with the goal of providing recommendations for refining MCI training protocols. By exploring the perceptions of MFRs who participated in scenario training, the aim of this study was to advance our understanding of the design of MCI scenario training and how to facilitate learning for MFRs.

## Methods

### Design

An inductive qualitative approach was chosen for the study.^21^ This approach ensured that the findings accurately reflected the participants’ experiences of MCI scenario training. To comprehensively understand potential differences and similarities in the current MCI scenario training curriculum, the study involved three EMS organisations and one search-and-rescue organisation tasked with responding to MCIs. Collectively, they represented four EU countries (Austria, Greece, Spain and Switzerland). During the study period, the included organisations were concurrently participating in the Horizon 2020 MED1stMR project.

As part of their involvement in MED1stMR, the organisations were tasked with carrying out MCI scenario training. These training exercises were designed in alignment with each organisation’s educational strategy, with the specific aim of equipping MFRs with the necessary skills to effectively manage MCIs. To account for the varying scales of the MCI scenario training across the organisations, an approach that embraced this diversity was adopted. Each organisation was responsible for designing its own training, allowing for the capture of a wide spectrum of current MCI scenario training methodologies being used to prepare MFRs. While the number of casualties varied between scenario trainings, so did the training focus. Three of the organisations (Austria, Greece and Switzerland) conducted multiple iterative exercises while the fourth organisation (Spain) conducted one MCI scenario training exercise. This variation is outlined in table 1.

**Table 1:** Overview of scenario training by organisation including number of interviewed participants

Participants (n)/number interviewed	Country	Organisation	Pedagogical methodology	Scenario training design	Training focus
37/5	Austria	EMS	Iterative exercises	Focused scenario	Crew resource management, medical treatment
26/10	Greece	Search-and-rescue	Iterative exercises	Multiple casualties	First triage, medical treatment
22/10	Spain	EMS	Single exercise	Multiple casualties	First triage, second triage
45/2	Switzerland	EMS	Iterative exercises	Focused scenario	Crew resource management, medical treatment
Total: 130/27					

EMSemergency medical service

The inductive qualitative approach of the study focused on capturing the diverse experiences of MFRs in current MCI scenario training. This diversity, inherent in the number of casualties and the scale of scenarios, was not viewed as a limitation. Instead, it was considered an integral part of the study design, contributing to the richness and diversity of the data. The study did not aim to standardise these experiences but to understand them in their varied contexts. Therefore, the insights derived were not influenced by the scale of the scenarios. This approach effectively mitigated any potential bias introduced by the differences in scenario scales. Prior to each exercise, a briefing session was conducted to outline the learning goals. After each exercise, debriefing sessions were held to discuss the exercise and participants’ performance.

### Patient and public involvement

Patients and/or the public were not involved in the design, or conduct, or reporting, or dissemination plans of this research.

### Participants

Between October 2021 and February 2022, 4 MCI scenario training exercises were conducted with the participation of a total of 130 MFRs. From this group, 27 MFRs were selected using a convenience sampling method (see table 1), ensuring a diverse representation of professional roles as suggested by previous research on data saturation.^22^ These selected MFRs, facilitated by their respective organisations, represented a wide spectrum of experience levels, ranging from extensive involvement in both MCI scenario training and real-world disaster response to those relatively new to these situations. The study encompassed participants from two key groups: EMS personnel and search-and-rescue volunteers. The specific demographics of the 27 selected MFRs, including their professional roles, gender distribution and years of experience, are detailed in table 2.

**Table 2:** Participant demographics

Sample characteristics	n	%
Gender		
Male	12	44
Female	15	56
Role		
Trainee	17	63
Instructor	10	37
Organisation		
EMS	17	63
Search-and-rescue	10	37
Country		
Austria	5	18.5
Greece	10	37
Spain	10	37
Switzerland	2	7.4
	**Mean**	**SD**
Experience as an MFR (years)	9.28	7.8
EMS	12	8,6
Search-and-rescue	4.65	2.4

The table details the participant group, gender distribution and mean years of experience (M), with SD indicating variability.

EMSemergency medical serviceMFRmedical first responder

### Data collection

To gain deeper insights into MFRs’ perceptions of their current MCI scenario training, the study used in-depth semistructured interviews.^23^,^25^ An interview guide, designed for a comprehensive exploration from individuals with firsthand experience, ensured consistency across researchers on the team before data collection.^25^ The interview guide presented consistent questions to each participant, establishing a uniform basis for data comparison and analysis.^23 24 26^ The questions were drafted based on a literature review that was aligned with the study’s aim. All authors were involved in the developing of the draft and throughout an iterative process the questions were discussed to ensure clarity and relevance. Ambiguities were addressed through the discussions, redundant questions were removed and new ones were added where necessary. For further validation, several experts in the field were also invited to review the questions. They assessed the relevance of the questions to the study’s aim, the clarity of the questions and the overall structure of the guide.

For organisations that conducted multiple iterative trainings, interviews were conducted after the final exercise. In contrast, for the organisation that conducted a single training exercise, interviews were conducted immediately after. This approach ensured that the interviews reflected the participants’ most recent experiences.^26^ The interviews lasted on average for 49 min and were conducted in private rooms for confidentiality. Interpreters assisted the researcher in conducting the interview in those instances when language barriers arose. The interpreters were native speakers involved with the MED1stMR project. All interviews were audio recorded and transcribed verbatim.

### Data analysis

Qualitative content analysis was used to process and analyse the data. The method was chosen as it allows for a deep understanding of the context in which the data was generated while also providing detailed insight into the data.^27 28^ Drawing on their previous experience and knowledge of qualitative content analysis, FS conducted the initial step of the analysis process. This encompassed a complete review of the transcribed interviews, which were transcribed using Microsoft Word, to achieve a comprehensive understanding of the material. The initial analysis was discussed with senior researcher LG to ensure the validity and reliability of the findings.

In the process of reviewing the transcribed interviews, direct quotations from the participants were identified as either ‘trainee’ or ‘instructor’ depending on their role during the scenario training in question. These quotations were selected to validate the analysis of the data, serving as illustrative examples that underpin the findings and interpretations.

Each transcribed interview was then repeatedly reviewed to identify meaning units in the form of direct quotations, aligning with the aim of the study. This process served as the basis for subsequent analysis. Each of the extracted meaningful units was initially condensed and then assigned a corresponding code. These codes were sorted and categorised using Microsoft Excel. Codes with similar content were organised to lay the groundwork for the reconstruction phase of the analytical process, leading to the formation of subcategories and categories. Throughout the analysis process, FS, as the lead researcher, continually engaged with the research team members (QN, AB, DS and LG). This iterative review and discussion of the findings were integral to minimising bias and ensuring a comprehensive and balanced interpretation of the data.^29^ An example of the analytical process is given in table 3.

**Table 3:** Example of qualitative content analysis following Graneheim and Lundman’s approach

Meaning unit	Condensed meaning unit	Code	Subcategory	Category
These practical sessions are great, but they could be even more useful if the other first responders as well, like police and firefighters, could also participate and be part of it…	These practical sessions are great, but they could be even more useful if the police and firefighters could also participate	More useful if police and firefighters could participate	Interagency collaboration	Training in a context mirroring real-world incidents

## Results

The results were derived through the analysis process, resulting in the identification of two categories and seven associated subcategories. These findings are summarised in table 4 and presented below in more detail.

**Table 4:** Overview of the results

Category	Subcategory
Training in a context mirroring real-world incidents	Conducting incident scene risk assessment Realistic representation in casualties Incorporating scenario variety into the curriculum Interagency collaboration Role alignment when training incident site management
Use of a pedagogical framework	Allowing for mistakes The importance of post-training evaluation

### Training in a context mirroring real-world incidents

This category highlights the significance of authentic training conditions for enhancing learning about MCI response. These conditions affect several key aspects of current MCI scenario training. The participants emphasised the importance of being able to practice disaster response and provide emergency medical care in a context that mirrors real-world incidents. However, they also acknowledged limitations in current MCI scenario training pertaining to low realism, the absence of interprofessional training and limited scenario variation.

#### Conducting incident scene risk assessment

Participants identified risk assessment during MCI scenario training as a key learning condition, given its importance in MFR responsibilities. They noted an increasing trend towards more dynamic situations, such as terrorism or violence incidents. To ensure safety in real-world incidents, participants emphasised the need for MCI scenario training that includes risk assessment practice. However, they found current training often lacks realistic elements like demolished cars or bystanders, limiting their ability to learn danger identification and control at the incident scene.

In the first of the scenarios it was like… it’s not realistic. Because [in real life] we’ll have the crowd, people asking questions, screaming, calling the police. You must be able to control all these things, the dangers around you. (Interviewee 16—Trainee)

#### Realistic representations in casualties

Participants in MCI scenario training stressed the importance of realistic casualty representations for learning to handle complex, life-threatening situations. They found a high degree of realism beneficial, as it mirrored real-world MCIs. This realism extended beyond the portrayal of individuals to include the authenticity of injuries. Participants expressed a preference for actors simulating casualties over manikins or static images, citing the increased realism and convincing nature of these portrayals. They highlighted that these realistic scenarios significantly enhanced their learning experience, as they were required to independently understand and respond to situations. The instances where actors portrayed the scenarios extremely realistically were particularly impactful, leading to significant learning gains.

The reason we conduct these scenario trainings is for the realism and the interaction with a patient. There are actors involved, some of whom portray the scenarios extremely realistically. These particular instances significantly enhance the learning experience. (Interviewee 1—Instructor)

#### Incorporating scenario variety into the curriculum

Participants highlighted the importance of diverse training scenarios for MFRs responding to MCIs, regardless of cause or scale. They stressed the need for a comprehensive frame of reference to effectively respond to real-world incidents, encompassing not just casualty numbers but also incident origins. However, they noted a limitation in current training: a focus on traffic-related incidents. Despite a desire for a broader range of scenarios in the curriculum, they found opportunities for training beyond traffic incidents were limited.

There are of course many possible disasters, be it landslide accidents and so on and so forth. But in school it’s primarily about traffic accidents, or the scenarios that we’ve played through are always actually centered around traffic accidents. We’ve discussed terrorist attacks with many casualties. But that was less part of the training and more like a sequence we looked at once. (Interviewee 21—Trainee)

#### Interagency collaboration

Participants acknowledged the complexities of MCIs and the value of collaboration with other responder services during MCI scenario training. A learning environment that enhanced mutual understanding of work methods and responsibilities was appreciated, as it benefited both their knowledge and casualty outcomes. Involvement of other first responder services was seen as an opportunity to share knowledge and strengthen bonds. However, they noted a limitation: a reluctance, possibly due to policy issues, to conduct exercises with other rescue services.

I generally see a bit of a problem with our provincial policy. There seems to be a reluctance to conduct [MCI scenario training] exercises with other rescue services, to learn from them, or to share knowledge. I think that is a shame, that you don’t work more closely with neighboring rescue services. (Interviewee 24—Instructor)

#### Role alignment when training incident site management

Analysis showed participants felt clinically prepared for MCIs but saw a gap in their experience with incident site management. With limited opportunities to learn from real incidents, they expressed a need for MCI scenario training to fill this gap. However, the participants noted a limitation in the training: a misalignment of roles and skills. This often resulted in the most medically trained individuals making decisions while those with less medical knowledge were given patient care roles. It was also observed that leadership roles, requiring more organisational than medical training, were often filled by individuals lacking in organisational skills.

It was often the case that the best medically trained people made decisions and assigned people to work. And the people who had the least medical knowledge were with the patients and worked there. That’s not the right way to do it, in my opinion. Someone who’s the head of a medical aid station or an operational area doesn’t necessarily need an insane amount of medical training, but they need organizational training. And a lot of people lack that. (Interviewee 25—Instructor)

### Use of a pedagogical framework

This category highlighted the importance of a pedagogical framework for learning during MCI scenario training. Without such a framework, the training lacks defined objectives and remains unevaluated. Therefore, participants emphasised the need for a structured review and evaluation process after each scenario. In addition, they pointed out the importance of creating a safe training environment that fosters learning.

### Allowing for mistakes

Participants acknowledged the risk and severity of mistakes during an MCI response, viewing MCI scenario training as a safe learning platform. This allowed for experimentation and understanding of actions’ consequences without fear of causing harm. However, they recognised that this controlled environment might not fully prepare them for real-world MCIs’ unpredictability and stress. They suggested incorporating real-world unpredictability into training scenarios to bridge this gap. Participants also raised concerns about the potential for false reassurance in an overly supportive environment. They noted that if the scenario design is too safe, it could inadvertently set them up for failure in a real MCI by not adequately preparing them for the stress and unpredictability of such situations. Therefore, striking a balance between providing a safe learning environment and simulating the challenges of real-world MCIs was considered by the participants to be essential. Instructors played a pivotal role in achieving this balance, guiding learners through mistakes while ensuring the training remained beneficial.

I felt quite confident because the instructors fostered a sense of confidence in us. They encouraged us to engage in training, to learn, to ask questions, and not to fear making mistakes. This is a controlled and safe environment, so we have that opportunity here. In a real-life scenario, we wouldn’t have this opportunity. So, we learn to feel okay with asking questions, making mistakes, and learning from them. (Interviewee 12—Trainee)

### The importance of post-training evaluation

Despite the existence of various performance evaluations within their service, participants perceived them as lacking in quality, often viewing them as informal discussions rather than in-depth assessments. They deemed performance evaluation during MCI scenario training essential for learning, driven by personal motivation to improve as an MFR. Participants acknowledged the criticality of error-free emergency medical care during an MCI. However, despite recognising the importance of comprehensive evaluations for learning and performance improvement, they found such assessments following scenario training to be limited.

We often have the problem as paramedics that we don’t get any feedback. And so, we have the problem that we don’t know; did we do that right, did we do that wrong? That’s the beauty of our profession but at the same time also the danger, that we don’t really have room for error. We work directly on patients, and we also work on critical patients. (Interviewee 23—Instructor)

## Discussion

This study highlights several crucial factors that could enhance learning in MCI scenario training. The integration of training in a context that mirrors real-world incidents, and the application of a pedagogical framework were the most prominent factors identified. However, the primary finding reveals a significant gap: current training programmes lack these vital areas. This absence largely impedes the learning conditions for MFRs. The study has identified these deficiencies, where the subsequent discussion will present recommendations for augmenting MCI scenario training from a learning perspective.

The findings of this study reveal that MFRs consider realistic representations of casualties as a crucial factor in MCI scenario training for managing complex, life-threatening situations. A heightened sense of realism in the scenarios was perceived to enhance the learning experience. This is consistent with the findings of MacLean *et al*, which highlight the positive correlation between the degree of perceived realism in training scenarios and the effectiveness of the learning outcomes.^30^ However, it is important to note that the precise degree to which realism influences learning outcomes is still the subject of research. It is, therefore, crucial to examine the threshold at which realism becomes sufficiently credible to enhance learning outcomes. This exploration could address the identified limitations related to realism that are present in current MCI scenario training.

MFRs acknowledged the need for a collaborative approach among diverse branches of first responder services. They emphasised the significance of collaborating with and incorporating personnel from both rescue services and law enforcement during MCI scenario training. This collaboration was seen by MFRs as an opportunity to mirror the conditions of a real-world disaster incident, share knowledge and foster stronger relationships among first responders. These findings align with those of Charman, which illustrate that training involving multiple first responder services not only strengthen collegial bonds but also enhances patient outcomes in real-world incidents.^31^ Our study not only underscores the importance of interagency collaboration and the development of incident site management skills but also suggests that these elements could be pivotal in shaping policies for MCI scenario training. Policy-makers and clinicians could consider our findings when designing training programmes to ensure they are as effective and realistic as possible.

In our study, MFRs highlighted the need to enhance their competencies in resource coordination and logistics management during large-scale emergencies. This is a key aspect of effective interagency collaboration. This emphasis on collaboration for competency development aligns with the findings of Berchtold *et al*, who highlight the role of interagency collaborations in promoting learning outcomes.^32^ Notably, the current MCI scenario training often lacks the inclusion of other first responder agencies as evidenced by the findings of this study.

Considering these findings, virtual reality (VR) environments with multiplayer functionality present a promising avenue. These environments can foster enhanced interorganisational collaboration, providing less resource-intensive training opportunities compared with live exercises.^33^ However, the implementation of such technology should consider organisational needs. Future research could explore how this technology can expand collaborative training opportunities and reduce costs.

MFRs perceived the performance evaluation within their service as deficient in both frequency and quality. This perception aligns with the findings of Morrison *et al*,^34^ who outlined how constraints in feedback delivery within a prehospital context can adversely affect patient outcomes. Echoing this, Wilson *et al* reported that MFRs find current feedback to be sporadic and express a need for more frequent feedback.^35^ As a result, a primary motivation for MFRs to participate in MCI scenario training, as revealed in our study, is the aspiration for a thorough post-training evaluation of their performance to sharpen their technical and non-technical skills. This motivation is consistent with Ryoo and Ha, who underscored the importance of debriefing in simulator-based learning as a crucial factor in facilitating learning.^36^

In addition to the traditional methods of post-training evaluation, there are innovative approaches being developed using technology. One such approach is the use of VR training solutions. As reported by Zechner *et al*, evaluations can be facilitated through internal dashboards, providing useful data for instructors to assess trainee performance.^37^ Insights from these dashboards assist instructors in identifying strengths and areas for improvement. This data-driven approach can contribute to the effectiveness of training programmes and improve learning outcomes. However, it is important to note that while VR training solutions offer many benefits, they also present new challenges. As reported by Xie *et al*, these challenges encompass maintaining the quality of the virtual training environment, resolving technical issues and making certain that the training is both effective and engaging to all users.^38^ Therefore, it is crucial to carefully consider these factors when implementing a VR training solution.

### Strengths and limitations of the study

This study used semistructured interviews conducted immediately following MCI scenario training. This approach facilitated the capture of MFRs most recent experiences with traditional MCI scenario training. The semistructured nature of the interviews allowed for flexibility in responses and the opportunity for follow-up questions, enabling a more in-depth understanding of the subject matter. This method provided a comprehensive exploration of the MFRs’ experiences and perspectives, thereby yielding valuable insights into the effectiveness and impact of MCI scenario training.

The study design incorporated both EMS personnel and search-and-rescue volunteers, providing a comprehensive understanding of the perspectives of MFRs and enriching insights into traditional MCI scenario training. This approach offered a broader view of the training’s impact and effectiveness across different groups of first responders. However, we acknowledge that a subset of our interviewees lacked direct experience in managing MCI in a real-world context. This may limit the direct applicability of our findings to real-world MCI responses. Nevertheless, we believe their insights still provide valuable perspectives for theoretical understanding and future research.

The heterogeneity of the participants, stemming from their diverse backgrounds and experiences, contributed to a richer data set, capturing a wide range of perspectives. This diversity, while enhancing the comprehensiveness of the study, also introduced variability in the data. Differences in education, training and previous experiences among the participants complicated the interpretation of the findings. Therefore, while the findings provide valuable insights into MCI scenario training, they should be interpreted with consideration of this variability.

While a consistent interview guide was used throughout the study, the use of interpreters for some interviews may have led to variations in question phrasing and comprehension, potentially influencing the responses and affecting the data collected. Additionally, the task of conducting MCI scenario training was assigned to the participating EMS. The inherent variability in each scenario’s design could have impacted the participants’ responses, introducing a limitation in the comparability of findings across different settings.

## Conclusions

This study not only reaffirms the merits of traditional MCI scenario training methods but also illuminates potential areas for enhancement. Advocating for more diverse and realistic scenarios, interagency collaboration, incident site management skills and improved post-training evaluation underscores the need for a more comprehensive and nuanced approach to MCI training. These results could also serve as an impetus for change, inspiring a shift in how MCI training is conceptualised and delivered.

Building on these insights, there is merit in investigating the capabilities of emerging technologies. Feedback from MFRs underscores the demand for more realistic and collaborative training scenarios. VR, with its capacity to generate immersive and interactive environments, could potentially meet these requirements. However, the introduction of new technologies also brings significant challenges, highlighting the necessity for additional research. It is important to acknowledge that the adoption of VR training involves financial considerations. Therefore, the cost-effectiveness of this approach requires further exploration. The choice of a training method should be informed by factors such as programme objectives, the target audience and available resources.

## Data Availability

No data are available.
