# Supplementary material for: Exploring medical first responders’ perceptions of mass casualty incident scenario training: a qualitative study on learning conditions and recommendations for improvement
Source: BMJ Open. 2024 Jul 11;14(7):e084925. doi: 10.1136/bmjopen-2024-084925 (PMC11243206; doi:10.1136/bmjopen-2024-084925)
Supplement: Supplementary file 1 [file bmjopen-2024-084925.reviewer_comments.pdf]

PEER REVIEW HISTORY

BMJ Open publishes all reviews undertaken for accepted manuscripts. Reviewers are asked to complete a checklist review form (**http://bmjopen.bmj.com/site/about/resources/checklist.pdf**) and are provided with free text boxes to elaborate on their assessment. These free text comments are reproduced below.

ARTICLE DETAILS

**TITLE (PROVISIONAL)**	Exploring Medical First Responders' Perceptions of Mass Casualty Incident Scenario Training: A Qualitative Study on Learning Conditions and Recommendations for Improvement
**AUTHORS**	Schulz, Fredrik; Nguyen, Quynh; Baetzner, Anke; Sjöberg, David; Gyllencreutz, Lina

VERSION 1 – REVIEW

**REVIEWER**	Anip Joshi<br>National Academy of Medical Sciences of Ukraine
**REVIEW RETURNED**	13-Feb-2024

**GENERAL COMMENTS**	This is a well conducted study and relevant to improve MCI scenario trainings.<br><br>1. In Introduction, it would be informative to the readers to mention about current status of MCI scenario trainings in 4 EU countries.<br><br>2. In Methods- design,<br>The authors have mentioned that two organisations conducted MCI scenario training involving multiple casualties while other two organisations engaged in smaller scale scenarios. How have the authors nullified the effect of this difference in scenario trainings in the study inference?<br><br>In Methods-data collection,<br>What was the methodology adopted to validate the semi- structured questionnaires?<br><br>In Methods- data analysis,<br>Which computer software was used for qualitative data analysis- generation of codes, categories,sub-categories, themes?<br><br>In Methods-<br>As there seems to be heterogeneity of study participants, the researchers have yielded rich data set with multiple themes. Was there "triangulation" implied to verify data saturation?<br><br>In Methods-,<br>Highlight briefing and debrief sessions if conducted as these are integral in MCI scenario trainings.<br><br>In Discussion,<br>Paragraph 5 , there is discussion about benefits of VR multiplayer technology which is not the result of this study. Reconsider the statement.

**REVIEWER**	Alena Lochmannová<br>University of West Bohemia in Pilsen, Department of paramedic science, medical diagnostics studies and public health
**REVIEW RETURNED**	20-Feb-2024

**GENERAL COMMENTS**	First of all, thank you very much for the opportunity to review this paper, which is very clearly written and presents a topic that is not only often on the fringes of interest, while it is a topic that is important not only for the direction of academic interests in the plane of science and research, but above all with the potential for societal benefit and application. I have some recommendations for authors that they may or may not consider:<br>- I would recommend defining who you mean by "first responders" - they may be individuals, first on the scene in the sense of professionals, but in some countries first responders are members of the public who receive training and the term "first" refers to the fact that they will be on the scene perhaps faster than emergency responders in some situations, given their location of residence (but they are not professionals).<br>- I recommend describing, at least briefly, how many and what content of scenarios the probands have been through<br>- I recommend justifying why you did not use a mixed research design - e.g. tracking error rate, time, subjective load (NASA TLS or others), etc. - we have done this in the past as part of an experiment on MCI on a sample of paramedics in virtual reality with very interesting results<br>- The section on VR is slightly confusing - have you or have you not used scenarios in VR? This is not clear from the article, I assume you mention VR as a possibility for further research (but these are already underway, our team has conducted several including a comparison of conventional and innovative training in VR targeted at triage for all forces - paramedics, police and firefighters) and you have added the section on VR more as a direction for further development, however at first glance it is confusing - note: AR has great potential here<br>- I would recommend giving more justification for why you have targeted search-and-rescue volunteers as well<br>- You state that the study covered four European countries, I would recommend explicitly stating which ones (the reason is that in many countries the approach and form of training of rescuers is different, which may be an influencing variable) - furthermore, I would be interested to know why you used translators? Why did the research team not use native speakers from the country?<br>- I would recommend keeping whether you used any software to analyse the qualitative data, and if so, which<br>- I recommend you describe more about the research sample and your choice of citations for the paper - is it merely illustrative? did you not create a model from your study?<br>- Comparing conventional training and scenarios - did you conduct a comparison with conventional training as part of your study? By what indicators?

**REVIEWER**	Rosel Tallach<br>Raigmore Hospital
**REVIEW RETURNED**	21-Feb-2024

**GENERAL COMMENTS**	The authors have undertaken an interview-based review of current training in pre-hospital response to mass casualty incidents. They have representation from a good spread of experience and

	background, overcoming language barriers and achieving a valuable insight as a result. They are to be congratulated. They have presented their findings well with good progression and easy to follow process in distilling themes and subthemes. They have made good use of the quotes from their interviewees to justify their themes. They have included an appropriate number of references on methodology.<br><br>Some simple corrections:<br>1. Introduction: Page 4, line 49 change promotes to promote<br>2. Page 10 line 33 "In this study, the observed MCI training opted" instead of "opts"<br><br>Request for editing<br>1. Under "allowing for mistakes" Could the authors discuss the issue of false reassurance by too supportive an environment. If the scenario design is too safe, then the training is setting them up to fail in a real MCI. This needs to be balanced against gaining confidence.<br>2. Page 10, the Discussion. Line 26 you mention the importance of pedagogical framework. While you included a paragraph and a quote on this in your results I did not see that you had identified how many interviews were conducted after a single exercise, and how many had been conducted after multiple iterative exercises. i.e. how many exercises were pedagogical. Perhaps a table in your results showing the types of MCI training, how many had been undertaken by each interviewee, and the various backgrounds of the interviewees. The demographics of your paper could be clearer.<br>2. page 10 line 33-50. This paragraph is poorly argued. The authors claim a causative relationship between realism of scenario and learning outcomes. All that is shown in their results is a causative relationship between realism and enjoyment – not behaviour change or improved performance in subsequent events.<br>The authors say that the MCI training they observed opted for low cost over actors. They then state that this affects the learning experience. "this study's findings align with this observation" is just repeating their own observation, of their own findings. This should be taken out.<br>"Our findings corroborate the research of MacLean et al who also found a positive correlation between realism and learning outcomes." This is not borne out by their results. The authors results indicate that more MFRs enjoyed the training in more realistic scenarios, but they have not shown a provable cause and effect with learning outcomes or improved performance. This needs to be reworded or show the results that can back this up. The paper as it is currently written has not established this causative relationship.<br>3. The authors go on to state that they have revealed that MFRs perception of realism may not fully reflect the competence and knowledge enhancement gained from training. Are they saying that the learning gained is not related to realism of scenario? This is in contradiction to their previous statement in point (2) above. Including references with an opposite view – i.e. learning is not related to realism - and including a non-biased critique of low vs high fidelity simulation would add depth and balance.<br>4. Page 10 lines 52-60. This is good<br>5. Page 11 line 18 The authors write, "However, our study raises new questions about how to fully leverage the benefit of VR" How? Where have you reported this in your results?<br>6. Would benefit from a discussion about cost. The fact these training exercises have happened at all (the authors mention that lack of exercise frequency was a theme in their interviews) is a cost

	to the organisations involved. The authors recommend more realism which is much more expensive in constrained healthcare systems when there is no provable improvement in behaviour or leadership. How does this translate to low-resource settings? What do the authors value more – pedagogical repetition or expensive high-fidelity? Either revise this conclusion or provide more evidence.<br>7. Page 11 lines 31-37 This is a very good point about the value of the feedback / debrief. This is where so many MCI training programmes fall down and you mention this in your results. This is perhaps more important than how realistic the actors are - it's the targeted feedback and opportunity for improvement and its applicability to the real world. This is also possible to deliver competently across any resource.<br>8. In limitations the authors should acknowledge that some (How many?) or their interviewees have never responded to an MCI in real life so the applicability to improving response in the real live scenario cannot be drawn.

VERSION 1 – AUTHOR RESPONSE

Reviewer #1	Original	Revised
Introduction: it would be informative to the readers to mention about current status of MCI scenario trainings in 4 EU countries.	As MCIs are predominantly infrequent events, many MFRs lack opportunities to learn from experiencing real-word catastrophic events firsthand (7). To emulate…	Beyond the foundational qualifications necessary for their roles, additional training is a pivotal in preparing MFRs for disaster response. Within the European Union (EU), each member state is individually responsible for organizing disaster response and implementing disaster response training programs (8, 9). As MCIs are predominantly infrequent events, many MFRs lack opportunities to learn from experiencing real-word catastrophic events firsthand (10). To emulate…
Methods – Design: The authors have mentioned that two organisations conducted MCI scenario training involving multiple casualties while other two organisations engaged in smaller scale scenarios. How have the authors nullified the effect of this difference in scenario trainings in the study inference?	[…] collectively representing four EU countries. Each participating organization was responsible for designing their respective training. Two organizations conducted MCI scenario training involving multiple casualties, emphasizing triage, communication, and logistics at the incident scene, while the other two organizations engaged in smaller-scale scenario training with a specific focus on trauma care provision.	Collectively, they represented four EU countries (Austria, Greece, Spain, and Switzerland). During the study period, the included organizations were concurrently participating in the Horizon 2020 MED1stMR project. As part of their involvement in MED1stMR, the organizations were tasked with carrying out MCI scenario training. These training exercises were designed in alignment with each organization's educational strategy, with the specific aim of equipping MFRs with the

		necessary skills to effectively manage MCIs. Table 1 outlines each organization's scenario training.
Methods – Data collection: What was the methodology adopted to validate the semi-structured questionnaires?	An interview guide, designed for a comprehensive exploration from individuals with firsthand experience, ensured consistency across researchers on the team before data collection (24). The standardized…	An interview guide, designed for a comprehensive exploration from individuals with firsthand experience, ensured consistency across researchers on the team before data collection (26). The interview guide presented consistent questions to each participant, establishing a uniform basis for data comparison and analysis (24, 25, 27). The questions were drafted based on a literature review, aligned with the study's aim. All authors were involved in the developing of the draft and throughout an iterative process the questions were discussed to ensure clarity and relevance. Ambiguities were addressed through the discussions, redundant questions were removed, and new ones were added where necessary. For further validation, several experts in the field were also invited to review the questions. They assessed the relevance of the questions to the study's aim, the clarity of the questions, and the overall structure of the guide.<br><br>Interviews were conducted…
Methods – Data analysis: Which computer software was used for qualitative data analysis- generation of codes, categories, sub-categories, themes?	This encompassed a complete review of the transcribed interviews to achieve a comprehensive understanding of the material.<br><br>…<br><br>Each of the extracted meaningful units was initially condensed and then assigned a corresponding code. Codes with	This encompassed a complete review of the transcribed interviews, which were transcribed using Microsoft Word, to achieve a comprehensive understanding of the material.<br><br>…<br><br>Each of the extracted meaningful units was initially

	similar content…	condensed and then assigned a corresponding code. These codes were sorted and categorized using Microsoft Excel. Codes with similar content…
Methods: As there seems to be heterogeneity of study participants, the researchers have yielded rich data set with multiple themes. Was there "triangulation" implied to verify data saturation?		**Note:** Thank you for your insightful comment. Our study indeed resulted in a rich data set with multiple categories and sub-categories due to the heterogeneity of the study participants. However, we did not employ triangulation in our study. We ensured data saturation by continuing the analysis until no new codes emerged from the semi-structured interviews, thus achieving inductive thematic saturation. This approach, while different from triangulation, allowed us to thoroughly explore the data and ensure that we captured all relevant categories and sub-categories.
Methods: Highlight briefing and debrief sessions if conducted as these are integral in MCI scenario trainings.	Two organizations conducted MCI scenario training involving multiple casualties, emphasizing triage, communication, and logistics at the incident scene, while the other two organizations engaged in smaller-scale scenario training with a specific focus on trauma care provision.	These training exercises were designed in alignment with each organization's educational strategy, with the specific aim of equipping MFRs with the necessary skills to effectively manage MCIs. Prior to each exercise, a briefing session was conducted to outline the learning goals. After each exercise, debriefing sessions were held to discuss the exercise and participants' performance. Table 1 outlines each organization's scenario training.
Discussion – Paragraph 5: there is discussion about benefits of VR multiplayer technology which is not the result of this study. Reconsider the statement.	Furthermore, VR environments with multiplayer functionality can enhance interorganizational collaboration. This technology provides less resource-intensive training opportunities compared to live exercises (34). However, our study raises new questions	Notably, the current MCI scenario training often lacks the inclusion of other first responder agencies as evidenced by the findings of this study. Considering these findings, VR environments with multiplayer functionality present a

	about how to fully leverage the benefits of VR multiplayer technology. Future research could explore how this technology can expand collaborative training opportunities and reduce costs. Additionally, it would be interesting to investigate how the threshold of realism impacts learning outcomes and whether there is an optimal level of realism for maximizing these outcomes.	promising avenue. These environments can foster enhanced interorganizational collaboration, providing less resource-intensive training opportunities compared to live exercises (36). However, the implementation of such technology should consider organizational needs. Future research could explore how this technology can expand collaborative training opportunities and reduce costs.
**Reviewer #2**	**Original**	**Revision**
I would recommend defining who you mean by "first responders" - they may be individuals, first on the scene in the sense of professionals, but in some countries first responders are members of the public who receive training and the term "first" refers to the fact that they will be on the scene perhaps faster than emergency responders in some situations, given their location of residence (but they are not professionals).	In the domain of emergency response, the occurrence of mass casualty incidents (MCIs) presents significant challenges for medical first responders (MFRs) (1, 2). Characterized by an…	In the domain of emergency response, the occurrence of mass casualty incidents (MCIs) presents significant challenges for medical first responders (MFRs) (1, 2). MFRs are defined as personnel who have received specialized training to respond to MCIs and are competent in providing immediate medical intervention and assistance in the affected area. The role encompasses both emergency medical service personnel (EMS) and trained volunteers (3). Characterized by an…
I recommend describing, at least briefly, how many and what content of scenarios the probands have been through		**Note:** We appreciate your recommendation. To address this, we have included a detailed table in the manuscript that outlines each scenario conducted by the participating organizations. This table provides information on the country, type of scenario training, and the focus of the training. We believe this addition will offer a comprehensive overview of the scenarios our probands have been through.
I recommend justifying why you did not use a mixed research design - e.g. tracking error rate,		**Note:** Thank you for your comment. We appreciate the suggestion to use a mixed

time, subjective load (NASA TLS or others), etc. - we have done this in the past as part of an experiment on MCI on a sample of paramedics in virtual reality with very interesting results		research design. Indeed, incorporating quantitative measures such as error rate, time, and subjective load could provide additional insights into the effectiveness of MCI scenario training. However, for this study, we chose an inductive qualitative approach to deeply understand the participants' experiences and perceptions of the training. This approach allowed us to capture rich, detailed data that might not be fully appreciated through quantitative measures.
The section on VR is slightly confusing - have you or have you not used scenarios in VR? This is not clear from the article, I assume you mention VR as a possibility for further research (but these are already underway, our team has conducted several including a comparison of conventional and innovative training in VR targeted at triage for all forces - paramedics, police and firefighters) and you have added the section on VR more as a direction for further development, however at first glance it is confusing - note: AR has great potential here	This emphasis on collaboration for competency development aligns with the findings of Berchtold et al., who highlight the role of interagency collaborations in promoting learning outcomes (33). Furthermore, VR environments with multiplayer functionality can enhance interorganizational collaboration. This technology provides less resource-intensive training opportunities compared to live exercises (34). However, our study raises new questions about how to fully leverage the benefits of VR multiplayer technology. Future research could explore how this technology…<br><br>*<br><br>[…] as a crucial factor in facilitating learning (37).<br><br><br>VR training solutions have the advantage of enabling thorough post-training evaluations. As reported by Zechner et al., evaluations can be facilitated through…	This emphasis on collaboration for competency development aligns with the findings of Berchtold et al., who highlight the role of interagency collaborations in promoting learning outcomes (35). Notably, the current MCI scenario training often lacks the inclusion of other first responder agencies as evidenced by the findings of this study. Considering these findings, VR environments with multiplayer functionality present a promising avenue. These environments can foster interorganizational collaboration, providing less resource-intensive training opportunities compared to live exercises (36). However, the implementation of such technology should consider organizational needs. Future research could explore how this technology…<br><br><br>*<br><br>[…] as a crucial factor in facilitating learning (39).

		In addition to the traditional methods of post-training evaluation, there are innovative approaches being developed using technology. One such approach is the use of VR training solutions. As reported by Zechner et al., evaluations can be facilitated through…
I would recommend giving more justification for why you have targeted search-and-rescue volunteers as well		**Note:** Thank you for your comment. As previously addressed, the scope of medical first responders in our study have been expanded in the Introduction to include both Emergency Medical Services (EMS) personnel and trained volunteers. We appreciate your attention to detail and hope this clarification addresses your concern.
You state that the study covered four European countries, I would recommend explicitly stating which ones (the reason is that in many countries the approach and form of training of rescuers is different, which may be an influencing variable).	To comprehensively understand potential differences and similarities in the current MCI scenario training curriculum, the study involved three EMS organizations and one search-and-rescue organization, collectively representing four EU countries.	To comprehensively understand potential differences and similarities in the current MCI scenario training curriculum, the study involved three EMS organizations and one search-and-rescue organization tasked with responding to MCIs. Collectively, they represented four EU countries (Austria, Greece, Spain, and Switzerland).
Furthermore, I would be interested to know why you used translators? Why did the research team not use native speakers from the country?	The interviews lasted on average for 49 minutes and were conducted in private rooms for confidentiality. Considering participants from various European countries, interpreters were present when language barriers arose. All interviews were…	The interviews lasted on average for 49 minutes and were conducted in private rooms for confidentiality. Interpreters assisted the researcher conducting the interview in those instances when language barriers arose. The interpreters were native speakers involved with the MED1stMR project. All interviews were…<br><br>**Note:** Thank you for your insightful comment. We

		appreciate your attention to detail. In response to your query about the use of translators, we would like to clarify that we indeed employed native speakers to provide translations when necessary during the interviews. This information has now been made explicit in the revised manuscript to avoid any confusion.
I would recommend keeping whether you used any software to analyse the qualitative data, and if so, which	This encompassed a complete review of the transcribed interviews to achieve a comprehensive understanding of the material.<br>…<br>Each of the extracted meaningful units was initially condensed and then assigned a corresponding code.	This encompassed a complete review of the transcribed interviews, which were transcribed using Microsoft Word, to achieve a comprehensive understanding of the material.<br>…<br>Each of the extracted meaningful units was initially condensed and then assigned a corresponding code. These codes were sorted and categorized using Microsoft Excel.
I recommend you describe more about the research sample and your choice of citations for the paper - is it merely illustrative? did you not create a model from your study?	Direct quotations from the participants are attributed as either "trainee" or "instructor", depending on their role during the scenario training in question.	In the process of reviewing the transcribed interviews, direct quotations from the participants were identified as either "trainee" or "instructor" depending on their role during the scenario training in question. These quotations were selected to validate the analysis of the data, serving as illustrative examples that underpin the findings and interpretations.
Comparing conventional training and scenarios - did you conduct a comparison with conventional training as part of your study? By what indicators?		**Note:** Thank you for your insightful question. In this study, we did not conduct a comparison with conventional training. The aim was to explore the viewpoints of MFRs on current MCI scenario training. Therefore, we did not use any indicators for comparison. We appreciate your feedback and

		will consider including comparative studies in our future research.
**Reviewer #3**	**Original**	**Revision**
Introduction: Page 4, line 49 change promotes to promote	Through a detailed examination of factors that promotes learning and the inherent limitations in current training strategies,	Through a detailed examination of factors that promote learning and the inherent limitations in current training strategies,
Introduction: Page 4, line 49 change promotes to promote	Through a detailed examination of factors that promotes learning and the inherent limitations in current training strategies,	Through a detailed examination of factors that promote learning and the inherent limitations in current training strategies,
Under "allowing for mistakes" Could the authors discuss the issue of false reassurance by too supportive an environment. If the scenario design is too safe, then the training is setting them up to fail in a real MCI. This needs to be balanced against gaining confidence.	[…] They suggested incorporating real-world unpredictability into training scenarios to bridge this gap. Participants emphasized cultivating an atmosphere accepting errors as part of learning, with instructors playing a pivotal role in fostering this atmosphere and guiding learners through mistakes.	[…] They suggested incorporating real-world unpredictability into training scenarios to bridge this gap. Participants also raised concerns about the potential for false reassurance in an overly supportive environment. They noted that if the scenario design is too safe, it could inadvertently set them up for failure in a real MCI by not adequately preparing them for the stress and unpredictability of such situations. Therefore, striking a balance between providing a safe learning environment and simulating the challenges of real-world MCIs was considered by the participants to be essential. Instructors played a pivotal role in achieving this balance, guiding learners through mistakes while ensuring the training remained beneficial.
Page 10, the Discussion. Line 26 you mention the importance of pedagogical framework. While you included a paragraph and a quote on this in your results I did not see that you had identified how many interviews were conducted after a single exercise, and how	To comprehensively understand potential differences and similarities in the current MCI scenario training curriculum, the study involved three EMS organizations and one search-and-rescue organization, collectively representing four EU countries. Each participating	To comprehensively understand potential differences and similarities in the current MCI scenario training curriculum, the study involved three EMS organizations and one search-and-rescue organization tasked with responding to MCIs. Collectively, they represented

many had been conducted after multiple iterative exercises. i.e. how many exercises were pedagogical. Perhaps a table in your results showing the types of MCI training, how many had been undertaken by each interviewee, and the various backgrounds of the interviewees. The demographics of your paper could be clearer.	organization was responsible for designing their respective training. Two organizations conducted MCI scenario training involving multiple casualties, emphasizing triage, communication, and logistics at the incident scene, while the other two organizations engaged in smaller-scale scenario training with a specific focus on trauma care provision.	four EU countries (Austria, Greece, Spain, and Switzerland). During the study period, the included organizations were concurrently participating in the Horizon 2020 MED1stMR project. As part of their involvement in MED1stMR, the organizations were tasked with carrying out MCI scenario training. These training exercises were designed in alignment with each organization's educational strategy, with the specific aim of equipping MFRs with the necessary skills to effectively manage MCIs. Table 1 outlines each organization's scenario training.
page 10 line 33-50. This paragraph is poorly argued. The authors claim a causative relationship between realism of scenario and learning outcomes. All that is shown in their results is a causative relationship between realism and enjoyment – not behaviour change or improved performance in subsequent events.<br>The authors say that the MCI training they observed opted for low cost over actors. They then state that this affects the learning experience. "this study's findings align with this observation" is just repeating their own observation, of their own findings. This should be taken out.<br>"Our findings corroborate the research of MacLean et al who also found a positive correlation between realism and learning outcomes." This is not borne out by their results. The authors results indicate that more MFRs enjoyed the training in more realistic scenarios, but they	In this study, the observed MCI training opts for cost-effective alternatives such as static images instead of actors to portray casualties. This choice influences MFRs perceptions of realism and, consequently, their learning experience. The study's findings align with this observation, indicating that MFRs favor the use of actors due to the enhanced sense of realism and conviction they provide. Our findings corroborate the research of MacLean et al. and Saus et al., who also found a positive correlation between perceived realism and learning outcomes (30, 31).	The findings of this study underscore that MFRs value realism as an important factor in MCI scenario training, asserting its role in enhancing learning. This aligns with findings of Maclean et al., which substantiates the correlation between the degree of perceived realism in training scenarios and the efficacy of the learning outcomes (32).

have not shown a provable cause and effect with learning outcomes or improved performance. This needs to be reworded or show the results that can back this up. The paper as it is currently written has not established this causative relationship.		
The authors go on to state that they have revealed that MFRs perception of realism may not fully reflect the competence and knowledge enhancement gained from training. Are they saying that the learning gained is not related to realism of scenario? This is in contradiction to their previous statement in point (2) above. Including references with an opposite view – i.e. learning is not related to realism - and including a non-biased critique of low vs high fidelity simulation would add depth and balance.	However, our study further extends this understanding by revealing that MFRs' perceptions of realism may not fully reflect the competence and knowledge enhancement gained from the training, a nuance not explored in previous studies. It is therefore crucial to examine the threshold at which realism becomes sufficiently credible to enhance learning outcomes. This exploration could address the identified limitations related to realism that are present in current MCI scenario training.	However, it is important to note that the precise degree to which realism influences learning outcomes is still the subject of research. It is therefore crucial to examine the threshold at which realism becomes sufficiently credible to enhance learning outcomes. This exploration could address the identified limitations related to realism that are present in current MCI scenario training.
Page 10 lines 52-60. This is good		**Note:** Thank you for your positive feedback.
Page 11 line 18 The authors write, "However, our study raises new questions about how to fully leverage the benefit of VR" How? Where have you reported this in your results?	This emphasis on collaboration for competency development aligns with the findings of Berchtold et al., who highlight the role of interagency collaborations in promoting learning outcomes (33). Furthermore, VR environments with multiplayer functionality can enhance interorganizational collaboration. This technology provides less resource-intensive training opportunities compared to live exercises (34). However, our study raises new questions about how to fully leverage the benefits of VR multiplayer technology. Future research could explore how this technology…	This emphasis on collaboration for competency development aligns with the findings of Berchtold et al., who highlight the role of interagency collaborations in promoting learning outcomes (35). Notably, the current MCI scenario training often lacks the inclusion of other first responder agencies as evidenced by the findings of this study. Considering these findings, VR environments with multiplayer functionality present a promising avenue. These environments can foster interorganizational collaboration, providing less resource-intensive training opportunities compared to live exercises (36). However, the

		implementation of such technology should consider organizational needs. Future research could explore how this technology…
Would benefit from a discussion about cost. The fact these training exercises have happened at all (the authors mention that lack of exercise frequency was a theme in their interviews) is a cost to the organisations involved. The authors recommend more realism which is much more expensive in constrained healthcare systems when there is no provable improvement in behaviour or leadership. How does this translate to low-resource settings? What do the authors value more – pedagogical repetition or expensive high-fidelity? Either revise this conclusion or provide more evidence.	However, it's crucial to underscore that while VR training formats appear promising, their long-term effectiveness remains uncertain and necessitates further research. Moreover, the selection of a training method should be guided by considerations such as program goals, target population, and available resources.	While VR training formats show promise, their long-term effectiveness remains to be determined. This point underscores the need for further research. Moreover, it should be noted that the implementation of VR training carries financial implications. Consequently, the cost-effectiveness of this approach warrants further investigation. The selection of a training method should be guided by considerations such as program goals, target population, and available resources.
Page 11 lines 31-37 This is a very good point about the value of the feedback / debrief. This is where so many MCI training programmes fall down and you mention this in your results. This is perhaps more important than how realistic the actors are - it's the targeted feedback and opportunity for improvement and its applicability to the real world. This is also possible to deliver competently across any resource.		**Note:** Thank you for your positive feedback. We agree that the value of feedback/debrief is a crucial aspect of MCI training programmes and we're glad that you recognized this in our work. We appreciate your insightful comment.
In limitations the authors should acknowledge that some (How many?) of their interviewees have never responded to an MCI in real life so the applicability to improving response in the real live scenario cannot be drawn.	The study design incorporated both emergency medical service personnel and search-and-rescue volunteers, providing a comprehensive understanding of the perspectives of MFRs and enriching insights into traditional	The study design incorporated both emergency medical service personnel and search-and-rescue volunteers, providing a comprehensive understanding of the perspectives of MFRs and enriching insights into traditional

	MCI scenario training. This approach offered a broader view of the training's impact and effectiveness across different groups of first responders.	MCI scenario training. This approach offered a broader view of the training's impact and effectiveness across different groups of first responders. However, we acknowledge that a subset of our interviewees lacked direct experience in managing MCI in a real-world context. This may limit the direct applicability of our findings to real-world MCI responses. Nevertheless, we believe their insights still provide valuable perspectives for theoretical understanding and future research.

VERSION 2 – REVIEW

**REVIEWER**	Alena Lochmannová<br>University of West Bohemia in Pilsen, Department of paramedic science, medical diagnostics studies and public health
**REVIEW RETURNED**	21-Apr-2024

**GENERAL COMMENTS**	Thank you for the opportunity to process a review on this very interesting article. I have already provided all my recommendations in the past, many of which have been incorporated by the authors, some of which have not. In general, however, I believe that the level of incorporation is satisfactory and the article can be published. I still have a problem with the use of the term 'first responder', where international practice of interpretation varies widely, which can be misleading; on the other hand, an honest reader will get a proper classification of who the authors are referring to from the text, so this reservation and the non-incorporation of some others does not prevent the publication of the text. I recommend the text for publication.
